# LRPL-VIO: A Lightweight and Robust Visual–Inertial Odometry with Point and Line Features

**DOI:** 10.3390/s24041322

**Published:** 2024-02-18

**Authors:** Feixiang Zheng, Lu Zhou, Wanbiao Lin, Jingyang Liu, Lei Sun

**Affiliations:** 1 College of Artificial Intelligence, Nankai University, Tianjin 300350, China; 2120210382@mail.nankai.edu.cn (F.Z.); zhoulu@nankai.edu.cn (L.Z.); 2120210419@mail.nankai.edu.cn (J.L.); 2 Shenzhen Research Institute, Nankai University, Shenzhen 518081, China; 2120160381@mail.nankai.edu.cn

**Keywords:** simultaneous localization and mapping (SLAM), visual–inertial odometry, point–line fusion

## Abstract

Visual-inertial odometry (VIO) algorithms, fusing various features such as points and lines, are able to improve their performance in challenging scenes while the running time severely increases. In this paper, we propose a novel lightweight point–line visual–inertial odometry algorithm to solve this problem, called LRPL-VIO. Firstly, a fast line matching method is proposed based on the assumption that the photometric values of endpoints and midpoints are invariant between consecutive frames, which greatly reduces the time consumption of the front end. Then, an efficient filter-based state estimation framework is designed to finish information fusion (point, line, and inertial). Fresh measurements of line features with good tracking quality are selected for state estimation using a unique feature selection scheme, which improves the efficiency of the proposed algorithm. Finally, validation experiments are conducted on public datasets and in real-world tests to evaluate the performance of LRPL-VIO and the results show that we outperform other state-of-the-art algorithms especially in terms of speed and robustness.

## 1. Introduction

State estimation is crucial for unmanned mobile platforms, especially when operating in GPS-denied areas. Simultaneous localization and mapping (SLAM) algorithms have the ability to provide real-time pose estimation and build consistent maps; thus, it is a crucial technique for robots, self-driving cars and augmented reality (AR) devices [1]. Pure visual SLAM algorithms [2,3,4], which use cameras as the sole sensor, are lightweight, low-cost, and have gained popularity over the past decade. However, they lack strong robustness because of sensitivity to illumination change and motion blur.

Many researchers have found that combining a camera with an inertial measurement unit (IMU) offers complementary advantages [5]. IMUs output high-frequency and biased inertial measurements while cameras produce images with rich information. Based on this, numerous visual–inertial odometry and SLAM systems are designed to obtain accurate and robust pose estimation. According to the estimation strategy, they can be divided into two categories: optimization-based methods and filter-based methods. The former constructs a factor graph with visual re-projection errors and IMU pre-integration errors to optimize poses and feature landmarks such as OKVIS [6] and VINS-Mono [7]. The computational load is managed using a sliding window and marginalization to achieve real-time performance. The latter holds a state vector which consists of body states (position, speed, orientation, and inertial biases) and a fixed number of history poses such as MSCKF [8] and HybVIO [9]. State propagation is finished on the basis of IMU kinematic model and visual update provides multi-frame constraints to produce an accurate trajectory. However, the aforementioned algorithms rely solely on points for visual constraints, which can lead to divergence or failure in low-texture environments.

As line features are abundant in human-made worlds, more and more VIO frameworks fuse both points and lines to improve their performance. PL-VIO [10] is the first optimization-based point–line visual–inertial odometry framework. Points, lines and IMU pre-integration terms are integrated into the optimization window to recover trajectories and scene appearances. Hence, it can outperform its predecessor VINS-Mono in some large difficult environments with severe sacrifice of running time. To speed up the processing of line features, the effect of the hidden parameters in the LSD algorithm [11] was studied in PL-VINS [12]. The authors modified a proper set of parameters to balance the speed and quality of line feature extraction in the original LSD for pose estimation tasks. In this way, PL-VINS is capable of outputting estimated poses in real-time. FPL-VIO [13] applied two methods to make the front end lightweight. It uses a fast line detection algorithm FLD [14] instead of LSD to extract line features and BRIEF descriptors [15] of midpoints to perform line matching, which greatly reduces the running time of the front end. The authors in [16] presented a similar solution, choosing EDlines [17] with gamma correction for rapid detection of long line features. They tracked a certain number of points on the line, instead of the entire segment, using the sparse KLT algorithm for line matching. As a result, the consumed time of line features in the front end is declined. However, the back end of these optimization-based methods is still a heavy module because of the repeated linearization of visual and inertial error terms, which becomes worse after fusing both point and line features [10].

Since filter-based methods avoid the re-linearization, they are considered to be more efficient [5]. Trifo-VIO [18] is a stereo point–line VIO algorithm based on MSCKF. After state propagation, both point and line features are used for visual update. However, the line features are parameterized using a 3D point and a normal vector in this system, which is an over-parameterized representation because a space line has only four degrees of freedom. Another MSCKF with lines framework is proposed in [19]. This system adopts the closest point method to represent line features and shows a good performance in real-world experiments. However, its front end uses LBD [20] to match line features; thus, its real-time performance is severely limited. A hybrid point–line MSCKF algorithm is proposed in [21]. Based on the sparse KLT algorithm, it tracks sampled points on the line between three consecutive frames in a predicting–matching way; thus, a new line can be recovered if the original one is lost. However, extra memories and operations are required in the hybrid framework since line feature landmarks are preserved in the state vector.

Most SLAM and odometry algorithms run on small-sized devices with limited available resources. How to provide accurate and high-frequency pose estimation with low computational consumption for multiple feature frameworks is still an open problem. To solve this, we propose a novel lightweight point–line visual–inertial odometry algorithm which can robustly track the poses of moving platforms. The main contributions of this paper are as follows:

- A novel filter-based point–line VIO framework with a unique feature selection scheme is proposed to produce high-frequency and accurate pose estimation results. The whole system is fast, robust, and accurate to work in complex environments such as weak texture and motion blur.
- A fast line matching method is proposed in order to decline the running time of the front end. The lines are matched using an endpoint–midpoint tracking way and a complete prediction–tracking–rejection scheme, which can ensure the matching quality with a fast speed.
- Validation experiments on public datasets and in real-world tests are conducted to evaluate the proposed LRPL-VIO. The results prove the better performance of LRPL-VIO compared with other state-of-the-art systems (HybVIO [9], VINS-Mono [7], PL-VIO [10], and PL-VINS [12]), especially in terms of speed and robustness.

The rest of this paper is organized as follows. Section 2 describes our filter-based point–line VIO system. The proposed fast line matching method is detailed in Section 3. The experiment results are explained and presented in Section 4. Finally, conclusion and future works are discussed in Section 5.

## 2. Filter-Based Point–Line Visual–Inertial Odometry

**Figure 1:**
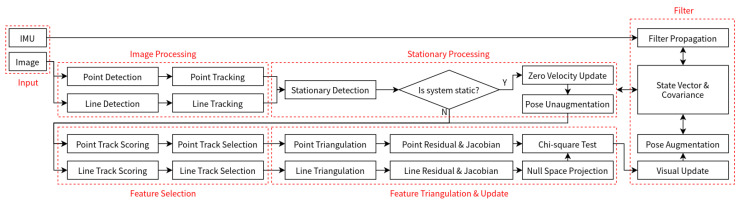
The working flowchart of LRPL-VIO.

While point-only visual–inertial odometry algorithms can produce accurate pose estimations in environments with constant illumination and rich texture, they often struggle, tending to diverge or fail in more challenging scenes. Fusing multiple features is a good solution, while the whole system becomes heavy. In this paper, we design a lightweight and efficient point–line VIO system based on HybVIO [9] to tackle this issue. The working flowchart of LRPL-VIO is shown in Figure 1.

### 2.1. State Definition

Similar to most filters derived from MSCKF [8], the state vector in our system consists of the body states and a window of past poses. At timestamp *k*, the state vector is constructed as:

(1) $$x_{k}={\left(p_{k}^{T},v_{k}^{T},q_{k}^{T},b_{k}^{T},\tau_{k},\Pi_{k}^{T}\right)}^{T},$$

where $p_{k}$ and $q_{k}$ denote the current pose of the body. $v_{k}$ is the velocity. And

(2) $$b_{k}={\left(b_{k}^{aT},b_{k}^{\omega T},diag{\left(T_{k}^{a}\right)}^{T}\right)}^{T}$$

is a vector related to inertial biases. Only the diagonal elements of $T_{k}^{a}$ are used for the multiplicative correction of the accelerometer. $\tau_{k}$ represents the IMU-camera time shift. A fixed-length window

(3) $$\Pi_{k}={\left(p_{k}^{(1)T},q_{k}^{(1)T},\ldots ,p_{k}^{(n_{a})T},q_{k}^{(n_{a})T}\right)}^{T}$$

holds $n_{a}$ poses of past moments.

### 2.2. Filter Propagation

The states are initialized as $m_{1|1}$ after obtaining the current orientation $q_{0}$ using the first inertial measurement. The initial covariance matrix $P_{1|1}$ are a diagonal matrix. The system are propagated using each subsequent inertial measurement as the prediction steps of the core filter:

(4) $$x_{k|k-1}=f_{k}\left(x_{k-1|k-1},\varepsilon_{k}\right),$$

where $\varepsilon_{k}∼N\left(0,Q_{k}\right)$ is the Gaussian process noise. This propagation is finished in discrete-time by a mechanization equation [22]:

(5) $$\left(\begin{array}{c} p_{k}\\ v_{k}\\ q_{k} \end{array}\right)=\left(\begin{array}{c} p_{k-1}+v_{k-1}\Delta t_{k}\\ v_{k-1}+\left[q_{k}\left({\tilde{a}}_{k}+\varepsilon_{k}^{a}\right)q_{k}^{*}-g\right]\Delta t_{k}\\ \Omega \left[\left({\tilde{\omega }}_{k}+\varepsilon_{k}^{\omega }\right)\Delta t_{k}\right]q_{k-1} \end{array}\right),$$

where $\Delta t_{k}$ is the current time increment. The biased inputs of gyroscope and accelerometer are calculated as ${\tilde{a}}_{k}=T_{k}^{a}a_{k}-b_{k}^{a}$ and ${\tilde{\omega }}_{k}=\omega_{k}-b_{k}^{\omega }$. $\varepsilon_{k}^{a}∼N\left(0,\Sigma_{k}^{a}\Delta t_{k}\right)$ and $\varepsilon_{k}^{\omega }∼N\left(0,\Sigma_{k}^{\omega }\Delta t_{k}\right)$ are i.i.d. Gaussian noises. g is the gravity vector. The rotation process represented by the quaternion is $q_{k}\left(\cdot \right)q_{k}^{*}$ and the quaternion is updated by the function $\Omega :R^{3}\to R^{4\times 4}$ [23]. The bias vector is propagated by

(6) $$\left(\begin{array}{c} b_{k}^{a}\\ b_{k}^{\omega }\\ T_{k}^{a} \end{array}\right)=\left(\begin{array}{c} \exp\left(-\alpha_{a}\Delta t_{k}\right)b_{k-1}^{a}+\epsilon_{k}^{a}\\ \exp\left(-\alpha_{\omega }\Delta t_{k}\right)b_{k-1}^{\omega }+\epsilon_{k}^{\omega }\\ T_{k-1}^{a} \end{array}\right),$$

where $\epsilon_{k}∼N\left(0,\frac{\sigma^{2}}{2\alpha }\left[1-\exp(-2\alpha \Delta t_{k})\right]\right)$ is modeled as the Ornstein–Uhlenbeck random walks [24] to better match the characteristics of the IMU sensor.

### 2.3. Image Processing

For points, we use the Good Features to Track (GFTT) algorithm [25] to extract new features and the sparse KLT optical flow algorithm [26] to perform feature tracking. The inertial measurements between consecutive frames are integrated to obtain the instant rotation. Initial values for the feature tracker, based on two-view geometry, could be obtained (See Equation (28)) and enhance tracking quality during rapid camera motions After all this, a hybrid 2-point [27] and 5-point [28] RANSAC method is performed to reject outliers.

For lines, we use the modified LSD algorithm [11,12] to detect new line segments and set a fixed threshold to abandon short lines. The line matching is finished using the proposed fast line matching method (See Section 3), which can greatly decrease the execution time of the front end and provide higher accuracy for our VIO system than the traditional descriptor-based method LBD [20].

### 2.4. Feature Selection

In addition to feature detection and matching, visual update in filter-based VIO methods is another time-consuming module. Paying more attention to the most informative features is an efficient way of decreasing computational load. Another novelty of the proposed LRPL-VIO is that we do not use all the tracked features (both points and lines) but a subset of them to perform visual updates.

For a visual feature *j*, its whole track is a set of pose indices $i=i_{min}^{j},\ldots ,i_{max}^{j}$ where $i_{min}^{j}$ denotes its first detection frame and $i_{max}^{j}$ denotes its last tracked frame. As the system moves, old poses are abandoned; thus, the oldest pose in the window denoted as b(i) may not be $i_{min}^{j}$ anymore. We use $b\left(i,j\right)=max(i_{min}^{j},b\left(i\right))$ to represent the oldest tracked frame in the window. Not all the measurements but a subset of them are used for triangulation and linearization:

(7) $$S\left(i,j\right)=\left\{b\left(i,j\right)\right\}\cup \{max\left(S\left(i^{\prime },j\right)\right)+1,\ldots ,i\},$$

where $i^{\prime }<i$ is the newest frame used in the last update. In a word, we always choose the freshest information for efficiency.

For a new received frame, we also select a subset of all available visual feature tracks (denoted as U(i)) to perform visual update at random from more-than-median ones

(8) $$\{j\in U\left(i\right)|L\left(i,j\right)>median_{U(i)}\left(L\left(i,\cdot \right)\right)\},$$

where the implementation of L(i,j) are different for points and lines in LRPL-VIO. For points, they are evaluated by the tracking length:

(9) $$L{\left(i,j\right)}_{point}=\sum_{l\in S(i,j)\setminus \{b(i,j)\}}{∥y_{l}^{j}-y_{l-1}^{j}∥}_{1},$$

where $y^{j}$ is the pixel coordinate. For lines, they are less sensitive to tracking length change than points. Thus, we use the frame number as the scoring policy:

(10) $$L{\left(i,j\right)}_{line}=i-max\left(S\left(i^{\prime },j\right)\right)-1,$$

which ensures the update accuracy even using a small number of line features.

### 2.5. Feature Triangulation and Update

The visual update is triggered track by track until the target number is reached:

(11) $$h_{k,j}\left(x_{k|k-1,j-1}\right)=\gamma_{k,j}∼N\left(0,\sigma_{visu}^{2}I\right)$$

with

(12) $$h_{k,j}\left(x\right)=d\left(r_{S}\left(\xi_{S}\left(x,{\tilde{y}}_{S}^{j}\right),x\right),{\tilde{y}}_{S}^{j}\right),$$

where

(13) $$\xi_{S}\left(x,{\tilde{y}}_{S}^{j}\right)=Tri\left(\Pi^{\left(S\right)},{\tilde{y}}_{S}^{j}\right)$$

denotes the triangulated landmark using its tracked feature measurements ${\tilde{y}}_{S}^{j}$. r(·) is the re-projection process and d(·) is the error calculation.

#### 2.5.1. Point Feature

The point error is the difference between the re-projected landmark and tracked measurements:

(14) $$h_{k,j}{\left(x\right)}_{point}=r_{S}\left(p_{S}\left(x,{\tilde{y}}_{S}^{j}\right),x\right)-{\tilde{y}}_{S}^{j},$$

where the point triangulation is the minimization process of the re-projection error

(15) $$RMSE_{j}\left(p_{S},x\right)=∥r_{S}\left(p_{S},x\right)∥$$

using the GN method. Since the Jacobian of $p_{S}$ with respect to x is available after the initial value is provided by a two-frame triangulation, the whole optimization process of Equation (15) needs to be differentiated to render the direct linearization of Equation (14) with respect to x

(16) $$h_{k,j}{\left(x\right)}_{point}\approx J_{h,k,j}\left(x_{0}\right)\left(x-x_{0}\right)+h_{k,j}{\left(x_{0}\right)}_{point},$$

which avoids the null space projection motion and can be used for visual update.

#### 2.5.2. Line Feature

The line error is defined as the distance between the endpoints of tracked measurements and the re-projected line:

(17) $$h_{k,j}{\left(x\right)}_{line}=\left[\begin{array}{c} d(r_{S}\left(l_{S}\left(x,{\tilde{y}}_{S}^{j}\right),x\right),{\tilde{y}}_{S,s}^{j})\\ d(r_{S}\left(l_{S}\left(x,{\tilde{y}}_{S}^{j}\right),x\right),{\tilde{y}}_{S,e}^{j}) \end{array}\right]$$

with

(18) $$d\left(l,e\right)=\frac{e^{T}l}{\sqrt{l_{1}^{2}+l_{2}^{2}}},$$

where $l=[l_{1},l_{2},l_{3}]$ is the re-projected line. For a space line representation, the Plücker coordinate [29] $L={\left[n^{T},d^{T}\right]}^{T}$ is used in our system. On the basis of two camera poses $\left(p_{j}^{\left(1\right)},q_{j}^{\left(1\right)},p_{j}^{\left(2\right)},q_{j}^{\left(2\right)}\right)$ and their corresponding measurements $\left(e_{s,j}^{\left(1\right)},e_{e,j}^{\left(1\right)},e_{s,j}^{\left(2\right)},e_{e,j}^{\left(2\right)}\right)$, we can obtain the dual Plücker matrix of a line feature [30] as

(19) $$L_{dual}=\pi^{\left(1\right)}\pi^{(2)T}-\pi^{\left(2\right)}\pi^{(1)T}=\left[\begin{array}{cc} {\left[d\right]}_{\times } & n\\ -n^{T} & 0 \end{array}\right],$$

where

(20) $$\pi =\left[\left(e_{s,j}^{w}-p_{j}\right)\times \left(e_{e,j}^{w}-p_{j}\right),-p_{j}\left(e_{s,j}^{w}\times e_{e,j}^{w}\right)\right]$$

are the measurement plane determined by two endpoints and the camera optical center. Triangulation depending on just two frames is not reliable enough; thus, we introduce a n-views method proposed in [31]. Specifically, for $n_{L}$ measurements of a line L, we stack all relevant planes:

(21) $$W={\left[\begin{array}{cccc} \pi^{(1)T} & \pi^{(2)T} & \ldots & \pi^{(n_{L})T} \end{array}\right]}^{T}$$

and perform singular value decomposition of Equation (21) as svd(W)=[s,d,v]. We can obtain two main planes $\pi_{1}$ and $\pi_{2}$ from the columns of v by checking two largest singular values. We use Equation (19) to obtain the initial value of L if the singular values are reasonable and perform a nonlinear optimization to further improve the accuracy of this triangulation. Based on the above methods, the linearization of Equation (17) is performed as

(22) $$h_{k,j}{\left(x,L\right)}_{line}\approx J_{h,k,j}\left(x_{0}\right)\left(x-x_{0}\right)+J_{h,k,j}\left(L_{0}\right)\left(L-L_{0}\right)+h_{k,j}{\left(x_{0},L_{0}\right)}_{line}$$

and the null space projection motion [19] is unavoidable for visual update because the feature positions are not maintained in the state vector.

### 2.6. Pose Augmentation and Stationary Detection

Every time a new camera frame is received, its predicted pose is inserted into the window and an old pose is removed. This process is performed as an EKF prediction step:

(23) $$x_{k+1|k}=A_{d}^{Aug}x_{k|k}$$

with

(24) $$A_{d}^{Aug}=\left(\begin{array}{ccc} I_{20} & \; & \\ I_{7} & \; & \\ \; & I_{7(d-1)} & \\ \; & \; & I_{7(n_{a}-d)} \end{array}\right).$$

The adjustment of *d* can be treated as an efficient strategy and we follow [9] to combine a fixed-size $n_{FIFO}$ with a Towers-of-Hanoi scheme:

(25) $$d_{i}=max\left(n_{FIFO},n_{a}-LSB\left(i\right)\right),$$

where LSB(i) is the least-significant zero bit index of *i*. Then the max stride of poses is exponentially increased and the update time of old and new poses are properly set to different frequencies.

When the moving platform stays still, the poses in the window are quickly be the same due to Equation (23), which makes the VIO unstable. Thus, an unaugmentation step is performed if a stationary signal is received as

(26) $$x_{k+1|k}={\left(A_{d}^{Aug}\right)}^{T}x_{k|k}+\left(\begin{array}{cc} 0_{dim(x)-7} & \\ \; & \varepsilon_{u} \end{array}\right),$$

which pops the new inserted frame and holds most of old poses. We judge the stationary condition by the maximum pixel change of tracked point features:

(27) $$m_{k}=\underset{j}{max}∥y_{k}^{j,L}-y_{k-1}^{j,L}∥<m_{min},$$

where $m_{min}$ is a fixed threshold. And a ZUPT of velocity [32] is also performed to correct the pose estimation results.

## 3. Fast Line Matching

The complex pixel distribution of line features makes their matching more challenging and time-consuming compared to point features. In this section, we propose a novel fast line matching method to break this bottleneck. An overview of our method is shown in Algorithm 1 and details are explained below.
**Algorithm 1** Fast Line Matching**Require:** $I_{1}$, $I_{2}$, $IMU_{1\to 2}$, K, $L_{1}$**Ensure:** $L_{2}$  1:$R_{21}\leftarrow Integrate\left(IMU_{1\to 2}\right)$  2:**for** $l_{i}\in L_{1}$ **do**  3:   $s_{i},m_{i},e_{i}\leftarrow Extract\left(l_{i}\right)$  4:   $Predict(R_{21},K,s_{i},m_{i},e_{i})$  5:   $s_{i}^{\prime },m_{i}^{\prime },e_{i}^{\prime }\leftarrow Track\left(I_{1},I_{2},s_{i},m_{i},e_{i}\right)$  6:   $l_{i}^{\prime }\leftarrow Outlier\;Reject\left(I_{1},I_{2},s_{i},m_{i},e_{i},s_{i}^{\prime },m_{i}^{\prime },e_{i}^{\prime }\right)$  7:   $L_{2}^{\prime }\leftarrow L_{2}^{\prime }\cup l_{i}^{\prime }$  8:**end for**  9:$L_{2}^{\prime }\leftarrow RANSAC\left(L_{1},L_{2}^{\prime }\right)$10:$L_{2}\leftarrow MatchAndRemoveShortLines\left(L_{2}^{\prime }\right)$11:**return***L*_2_

**Extraction:** For each line feature, tracking is focused on its two endpoints and midpoint, rather than the entire line or other sampled points. In other words, for *n* line features, we have 3n points in total.

**Prediction:** To counteract aggressive motions, inertial measurements between two camera frames are used to determine the initial positions of the points for tracking. Specifically, for two consecutive frames, $I_{1}$ and $I_{2}$, a point transformation between them is:

(28) $$\lambda_{2}K^{-1}v_{2}=\lambda_{1}R_{21}K^{-1}v_{1}+t_{21},$$

where $v_{1}$ and $v_{2}$ are pixel coordinates of the same point in these frames. $\lambda_{1}$ and $\lambda_{2}$ are the corresponding depth measurements. K is the intrinsic matrix which is considered as a static variable. The pose between $I_{1}$ and $I_{2}$ is represented by $R_{21}$ and $t_{21}$. By taking the assumption that the translation $t_{21}$ between two consecutive frames is small enough to be ignored, $\lambda_{1}$ and $\lambda_{2}$ can be removed from Equation (28). Thus, a simplified version is:

(29) $$v_{2}=KR_{21}K^{-1}v_{1}.$$

We obtain the rotation $R_{21}$ through gyroscope measurements integration and then the predicted positions of the points using Equation (29).

**Tracking:** After the above stages, the line matching task becomes the tracking of the points, which is finished based on the photometric invariance assumption in LRPL-VIO. Take a single line endpoint as an example. With its original pixel coordinate (x,y) in $I_{1}$, our idea is to find the target pixel coordinate (x+dx,y+dy) in $I_{2}$ to satisfy Equation (30):

(30) $$I_{1}\left(x,y\right)=I_{2}\left(x+dx,y+dy\right),$$

where $I_{i}\left(a,b\right)$ is the photometric value of the pixel (a,b) in $I_{i}$. Apparently we can not obtain (dx,dy) using one equation; thus, another assumption that the movements of all pixels in a local window are the same is applied. That is, we have

(31) $$\left\{\begin{array}{c} I_{1}\left(x_{1},y_{1}\right)=I_{2}\left(x_{1}+dx,y_{1}+dy\right)\\ I_{1}\left(x_{2},y_{2}\right)=I_{2}\left(x_{2}+dx,y_{2}+dy\right)\\ \ldots \\ I_{1}\left(x_{w},y_{w}\right)=I_{2}\left(x_{w}+dx,y_{w}+dy\right) \end{array}\right.$$

for all *w* pixels in the window. To solve Equation (31), a nonlinear optimization problem is constructed:

(32) $$dx,dy=arg\min_{dx,dy}{∥g\left(dx,dy\right)∥}^{2},$$

where

(33) $$g\left(dx,dy\right)=\sum_{i=1}^{w}\left(I_{1}\left(x_{i},y_{i}\right)-I_{2}\left(x_{i}+dx,y_{i}+dy\right)\right).$$

Equation (32) is a typical least squares problem and can be solved in an iterative way with the initial values provided by Equation (29). In addition, the image pyramids are introduced to improve the tracking quality.

**Outlier Rejection:** As long as the points of a line feature are tracked, we first check the average photometric values of two endpoints. In other words, an endpoint track is considered as an inlier if

(34) $$\Delta \bar{I}=\frac{1}{w}\sum_{i=1}^{w}\left(I_{1}\left(x_{i},y_{i}\right)-I_{2}\left(x_{i}+dx,y_{i}+dy\right)\right)<\varepsilon_{I},$$

where $\varepsilon_{I}$ is the threshold. However, Equation (34) is not enough to reject outliers when there is a large repeated texture area in the image. For this reason, an angle variation check is also performed if both two endpoints passed Equation (34). Namely, if a line matching pair $\left[\left(s_{i},e_{i}\right),\left(s_{i}^{\prime },e_{i}^{\prime }\right)\right]$ meets

(35) $$\Delta \theta_{i}=\left|\theta_{i}-\theta_{i}^{\prime }\right|<\varepsilon_{\theta },$$

where $\theta_{i}$ and $\theta_{i}^{\prime }$ are the angles of the line in consecutive frames, $\left(s_{i}^{\prime },e_{i}^{\prime }\right)$ is seen as a candidate line.

Generally, endpoints have the potential to move out of view or be tracked unsuccessfully. Hence, after obtaining the first batch of candidate lines by checking endpoints, we take tracked midpoints as new endpoints of the line features which failed to pass the above tests. For example, if $\left[\left(s_{i},e_{i}\right),\left(s_{i}^{\prime },e_{i}^{\prime }\right)\right]$ is not an acceptable tracking result, it will be replaced by $\left[\left(s_{i},m_{i}\right),\left(s_{i}^{\prime },m_{i}^{\prime }\right)\right]$ or $\left[\left(m_{i},e_{i}\right),\left(m_{i}^{\prime },e_{i}^{\prime }\right)\right]$. Certainly, the replaced line pairs have to satisfy both Equations (34) and (35). This scheme is able to improve the tracking length of line features with no additional sampled points. Finally, an 8-point RANSAC is performed to further reject outliers in these candidates.

**Matching:** After all this, we build matched line features through connecting the reserved endpoints and remove short ones which are useless for pose estimation.

## 4. Experiments

### 4.1. Dataset and Evaluation

To validate the necessity of fusing point–line features and the performance of our LRPL-VIO in different scenes, we conduct various experiments on three public academic datasets (EuRoC [33], UMA-VI [34], and VIODE [35]) and a collected real-world dataset. Four state-of-the-art algorithms (point-based VINS-Mono [7] and HybVIO [9], point–line-based PL-VIO [10] and PL-VINS [12]) are selected for comparison.

For the evaluation criteria, we choose the root mean square error (RMSE) of the absolute trajectory error (ATE) to test the estimation accuracy of different algorithms. For the EuRoC, VIODE and our collected dataset which provide groundtruth poses during the whole running process, we use the evo [36] toolbox to compute RMSE ATE between the whole estimated trajectory and groundtruth poses. For the UMA-VI dataset whose groundtruth poses are available at the start and end segments of the whole running process, we use their python tool to compute RMSE ATE between these segments of the estimated trajectory and the ground truth poses (the alignment error [34,37]). And we report the average value of five times.

A desktop computer with an Intel Core i7-9750H processor @2.60GHz and 15.5 GB RAM is used as the main experiment platform running Ubuntu 18.04 with ROS melodic.

### 4.2. Accuracy

In this subsection, we conduct an accuracy experiment on the EuRoC [33] dataset. It is made by a micro aerial vehicle (MAV) in three different indoor scenes. Sequences in each scene are divided into three modes: easy, medium, and difficult, according to the image quality and MAV motion speed. The results are shown as follows.

#### 4.2.1. Ablation Experiment

In order to validate the effectiveness of our LRPL-VIO with point–line fusion, fast front end and feature track selection, we first conduct an ablation experiment on five sequences of EuRoC dataset including MH_02_easy, MH_03_medium, MH_05_difficult, V1_03_difficult, and V2_02_medium. We replace the fast line matching method with the PL-VINS LBD matching module in our system (denoted as LRPL-VIO (LBD)) for matching comparison. And the line feature selection module is disabled (denoted as LRPL-VIO (All Line Track)) to prove its necessity. The results are shown in Table 1.

First, it can be seen from Table 1 that the point–line fusion strategy could bring more visual constraints for the VIO system; thus, LRPL-VIO could produce more accurate trajectories than the point-only HybVIO (with 11% enhance on the average). Second, the proposed fast line matching method could finish line matching more efficiently than LBD with higher matching quality (LRPL-VIO obtains lower RMSE ATE than LRPL-VIO (LBD) on all five sequences) and less running time (See Table 6). Finally, the feature track selection scheme avoids using all tracked line features and their updated measurements; thus, the pose estimation accuracy could be guaranteed (with 2% enhance on the average) even using a small numbers of features (5 successful line updates at most for one frame in our implementation).

#### 4.2.2. Accuracy Experiment

We use all 11 sequences on the EuRoC dataset to test the pose estimation accuracy of LRPL-VIO and compare it with four SOTA open-source algorithms. The results are shown in Table 2.

Compared with two point-only methods VINS-Mono and HybVIO, LRPL-VIO outperforms them on most sequences because of successful point–line fusion. Using visual constraints from various features, visual–inertial navigation systems could perform pose estimation more accurately. The average RMSE of LRPL-VIO is more than 10% lower than them. With improved line matching quality using the proposed method and feature selection scheme, line features could be used in LRPL-VIO in a more efficient way. Thus, compared with the LBD-based PL-VIO and PL-VINS, we outperform them with more than 7% lower average RMSE and less computational resource consumption (See Table 6).

### 4.3. Robustness

To further validate the robustness of the proposed LRPL-VIO, we select some challenging sequences from the following two datasets:

The UMA-VI dataset [34] is recorded by a custom handheld visual–inertial sensor suite. The images recorded in different scenes are severely affected by many challenging factors including low texture, illumination change, sun overexposure, and motion blur, which makes it a difficult dataset for VIO algorithms.

The VIODE dataset [35] is recorded by a simulated unmanned aerial vehicle (UAV) in dynamic environments. The novelty of this dataset is that the UAV navigates the same path in four sub-sequences (none, low, mid, high) of each scene, and the only difference between them is the number of dynamic objects.

The sequence features are listed in Table 3 and the results are shown in Table 4.

**Figure 2:**
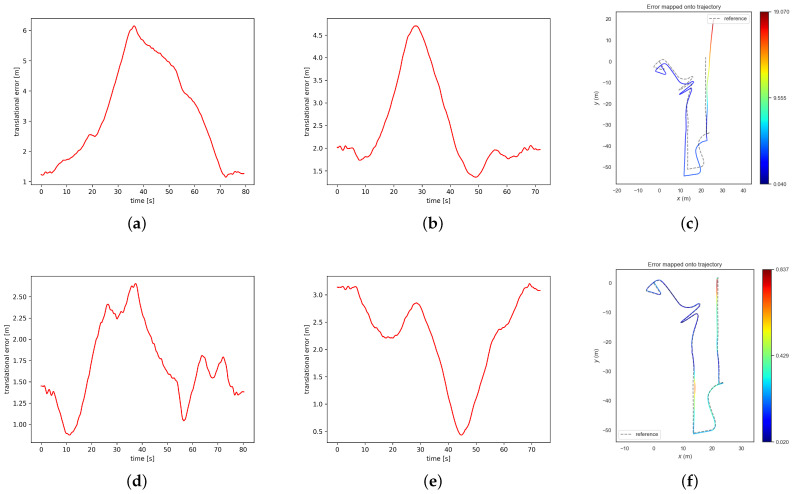
The pose estimation error of PL-VINS and LRPL-VIO on the UMA-VI and VIODE dataset. (**a**) The alignment error of PL-VINS in class_csc2. (**b**) The alignment error of PL-VINS in parking_csc2. (**c**) The RMSE ATE of PL-VINS in cd3_high. (**d**) The alignment error of LRPL-VIO in class_csc2. (**e**) The alignment error of LRPL-VIO in parking_csc2. (**f**) The RMSE ATE of LRPL-VIO in cd3_high.

Effective point–line fusion strategy could improve the robustness of visual–inertial odometry algorithms. From Table 4, we can see that PL-VINS and LRPL-VIO can perform successful pose estimation on all these challenging sequences. However, we show a better performance with a lower error on each sequence, which validates the better robustness of LRPL-VIO. We also provide the alignment error figures and heat maps of estimated trajectories of PL-VINS and LRPL-VIO in Figure 2. For the alignment error figures, the smaller the translational error is, the better accuracy the VIO could provide. For the heat maps, we could focus on the difference between the estimated trajectory and groundtruth poses, which is marked in different colors. Based on this, Figure 2 can validate the better robustness of LRPL-VIO than PL-VINS on the other hand.

### 4.4. Real-World Performance

**Figure 3:**
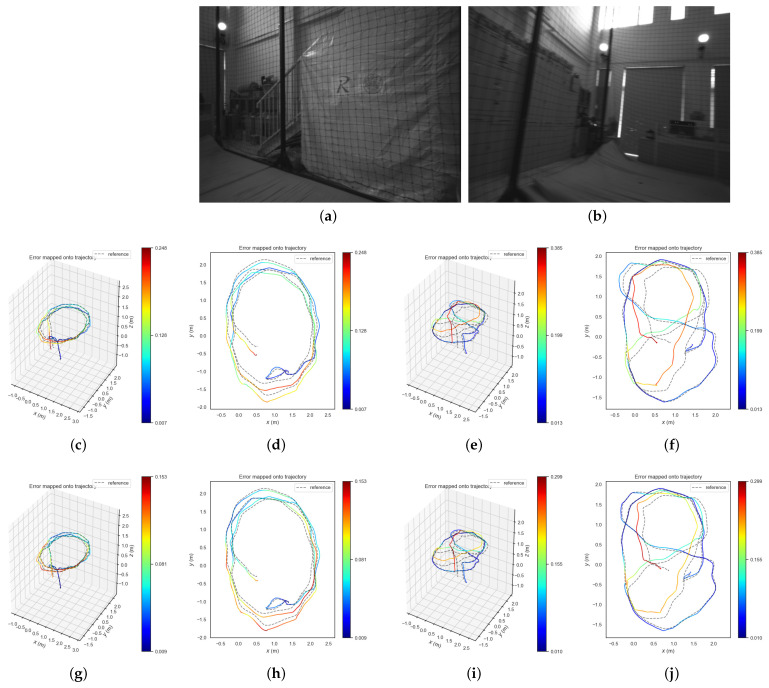
The figures of real-world experiments. (**a**) An example image of sequence Lab_Normal. (**b**) An example image of sequence Lab_FastRotation. (**c**) The 3D error map of HybVIO in Lab_Normal. (**d**) The X-Y plane of 3D error map of HybVIO in Lab_Normal. (**e**) The 3D error map of HybVIO in Lab_FastRotation. (**f**) The X-Y plane of 3D error map of HybVIO in Lab_FastRotation. (**g**) The 3D error map of LRPL-VIO in Lab_Normal. (**h**) The X-Y plane of 3D error map of LRPL-VIO in Lab_Normal. (**i**) The 3D error map of LRPL-VIO in Lab_FastRotation. (**j**) The X-Y plane of 3D error map of LRPL-VIO in Lab_FastRotation.

To test the performance of LRPL-VIO in real-world applications, we collected a custom dataset in a challenging indoor scene. A sensor suite with a Intel Realsense D455 camera (gray image, 30 Hz) and a Xsens MTi-680G IMU (inertial measurement, 200 Hz) is used as the collection platform. Two motion modes (normal and fast rotation) are applied to produce different evaluation sequences, which are shown in Figure 3a,b. The results are shown in Table 5.

From Table 5, it can be seen that LRPL-VIO could perform pose estimation more accurately than HybVIO in the experiments. The RMSE ATE of LRPL-VIO is 35.4% lower in Lab_Normal and 26.5% lower in Lab_FastRotation. Fusing various features could bring more constraints; thus, the whole estimated trajectories of LRPL-VIO are closer to groundtruth poses. And Figure 3c–j could validate this more intuitively.

### 4.5. Runtime

To evaluate the real-time performance of LRPL-VIO, we divide it into three main modules including point processing (front end), line processing (front end), and VIO (back end) for convenience of comparison with PL-VIO and PL-VINS. And the MH_04_difficult sequence of EuRoC dataset is used to conduct this test. The results are shown in Table 6.

As shown in Table 6, the time-consuming LBD and the heavy optimization back end are the most time-consuming module of PL-VIO and PL-VINS. In contrast, the proposed fast line matching method in Section 3 brings our system high efficiency. The execution time of line detection and tracking process of LRPL-VIO is much less than them. In addition, our core pose estimation scheme is an efficient EKF with a unique feature selection scheme, which ensures that our total processing speed of a single frame is nearly three times faster than PL-VINS.

## 5. Conclusions and Future Work

In this paper, a novel point–line visual–inertial odometry is proposed to address positioning issues in complex environments such as weak texture and dynamic features. The short runtime of feature correspondence is maintained by a fast line matching method; thus, the whole system can work at a high frequency. A line feature selection scheme is utilized to further improve the efficiency of the core filter. Validation experiments on the EuRoC, UMA-VI, and VIODE dataset have shown the better performance and efficiency of our system against other SOTA open-source algorithms (HybVIO [9], VINS-Mono [7], PL-VIO [10], and PL-VINS [12]). In the future, we will try to introduce the structural constraints of 3D line features and plane features to further improve the accuracy. 

## Figures and Tables

**Table 1:** The results of the ablation experiment, which is evaluated using RMSE ATE in meter.

	HybVIO	LRPL-VIO (LBD)	LRPL-VIO (All Line Track)	LRPL-VIO
MH_02_easy	0.213	0.186	0.160 ^1^	0.178 ^2^
MH_03_medium	0.319	0.315	0.307 ^2^	0.281 ^1^
MH_05_difficult	0.368	0.362	0.355 ^1^	0.358 ^2^
V1_03_difficult	0.110 ^1^	0.140	0.133	0.118 ^2^
V2_02_medium	0.127	0.077 ^2^	0.080	0.076 ^1^
Mean	0.227	0.216	0.207 ^2^	0.202 ^1^
Enhance	11%	6%	2%	-

^1^ means the best while ^2^ means the second best.

**Table 2:** The results of the pose estimation accuracy test, which is evaluated using RMSE ATE in meter.

	PL-VIO	VINS-Mono	HybVIO	PL-VINS	LRPL-VIO
MH_01_easy	0.136 ^1^	0.155 ^2^	0.288	0.157	0.212
MH_02_easy	0.141 ^1^	0.178	0.213	0.170 ^2^	0.178
MH_03_medium	0.264	0.194 ^1^	0.319	0.227 ^2^	0.281
MH_04_difficult	0.363	0.364	0.218 ^1^	0.275 ^2^	0.218 ^1^
MH_05_difficult	0.276 ^1^	0.303	0.368	0.288 ^2^	0.358
V1_01_easy	0.083	0.089	0.084	0.075 ^2^	0.053 ^1^
V1_02_medium	*	0.112	0.104 ^2^	0.123	0.088 ^1^
V1_03_difficult	0.199	0.187	0.110 ^1^	0.182	0.118 ^2^
V2_01_easy	0.088	0.087	0.057 ^1^	0.081	0.058 ^2^
V2_02_medium	0.135	0.152	0.127	0.124 ^2^	0.076 ^1^
V2_03_difficult	0.281	0.293	0.127 ^1^	0.210	0.144 ^2^
Mean	0.197	0.192	0.183	0.174 ^2^	0.162 ^1^

* means failure. ^1^ means the best while ^2^ means the second best.

**Table 3:** The features of the selected challenging sequences.

Dataset	Sequence	Features
UMA-VI	class_csc2	low texture, indoor–outdoor change
	parking_csc2	low texture, dark scene, illumination change
	third_floor_eng	low texture, illumination change, fast motion
VIODE	cd3_high	dynamic objects
	cn3_high	dark scene, dynamic objects

**Table 4:** The results of the robustness experiment. For evaluation, the alignment error in meter is calculated on the UMA-VI dataset and the RMSE ATE in meter is calculated on the VIODE dataset.

	PL-VIO	VINS-Mono	HybVIO	PL-VINS	LRPL-VIO
class_csc2	*	*	2.491 ^2^	3.507	2.161 ^1^
parking_csc2	*	7.869	5.101	4.216 ^2^	2.479 ^1^
third_floor_eng	*	*	*	14.511 ^2^	4.929 ^1^
cd3_high	1.358	1.653	0.619 ^2^	5.946	0.391 ^1^
cn3_high	0.861 ^2^	0.639 ^1^	0.971	1.225	0.864

* means failure. ^1^ means the best while ^2^ means the second best.

**Table 5:** The results of the real-world experiments, which is evaluated using RMSE ATE in meter.

Sequence	HybVIO	LRPL-VIO	Enhance
Lab_Normal	0.130	0.084 ^1^	35.4%
Lab_FastRotation	0.215	0.158 ^1^	26.5%

^1^ means the best.

**Table 6:** The results of the runtime analysis, which is evaluated using millisecond.

	Point Processing	Line Processing	VIO	Sum
PL-VIO	22.1	115.2	28.7	166.0
PL-VINS	15.0	32.0	46.0	93.0
LRPL-VIO	9.5	8.8	12.2	30.5

## Data Availability

Data sharing is not applicable.
